# Supplementary material for: MetaRibo-Seq measures translation in microbiomes
Source: Nat Commun. 2020 Jun 29;11:3268. doi: 10.1038/s41467-020-17081-z (PMC7324362; doi:10.1038/s41467-020-17081-z)
Supplement: Supplementary file 10 — Supplementary Data 7 [file 41467_2020_17081_MOESM10_ESM.zip › File2/Confidence_High_Taxonomy/100195_out.krona.html]

Javascript must be enabled to view this page.

members
magnitude
magnitudeUnassigned
count
unassigned
taxon
rank

100195\_out

3

3
2
superkingdom

201174
3
phylum

class
3
1760

2037
1
order

2049
1
family

genus
1654
1


SRS104830\_contig\_number\_contig-100\_4056.4056
1
712118
species

order
2
85007

2
1653
family

1716
2
genus

species

SRS063215\_contig\_number\_contig-100\_5476.103459
43768
1

species

SRS104921\_contig\_number\_19156
1
61592
